# Electrorheological Fluids Based on Porous Carboxyl-Functionalized Polytriphenylamines

**DOI:** 10.1021/acsapm.4c02469

**Published:** 2025-01-25

**Authors:** Ozlem Erol, Ulzhalgas Karatayeva, Charl F. J. Faul

**Affiliations:** † Chemistry Department, Science Faculty, Gazi University, 06560 Ankara, Turkey; ‡ School of Chemistry, University of Bristol, Bristol BS8 1TS, U.K.

**Keywords:** conjugated microporous polymers, electrorheological fluids, polytriphenylamine, stimuli-responsive fluids, surface functional groups

## Abstract

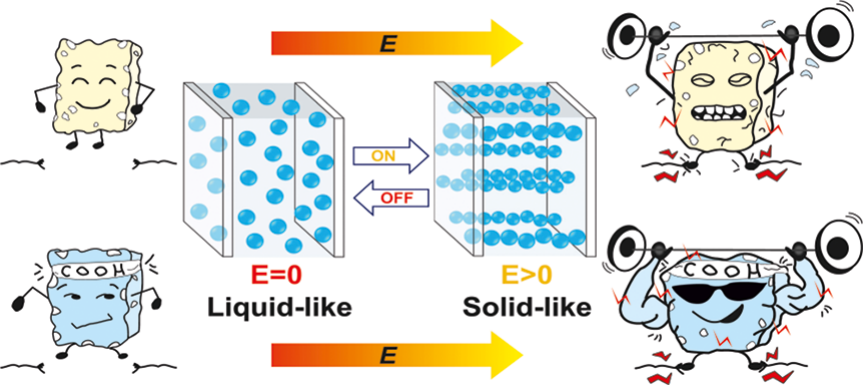

Electrorheological fluids (ER) make up a class of smart materials that are distinguished by their capacity to alter their rheological characteristics in a controlled and reversible manner in response to an externally applied electric field (*E*). As an inherent polarizable anhydrous ER-active material, polyaniline (PAni)-based materials are among the most frequently utilized ER-active materials. However, PAni can only be used as an ER-active material after carefully adjusting its conductivity to an appropriate range. Three-dimensional (3D) conjugated microporous polymer (CMP) analogs of PAni have a nitrogen-rich porous and hierarchical structure, low density, and appropriate conductivity that can be used to explore ER performance and dispersion stability. In this study, a carboxylic acid functionalized version with a greater polar porous surface was designed to obtain higher polarizability and appropriate conductivity without the need for any dedoping process and thus enhanced ER performance. For this purpose, polytriphenylamine (PTPA) and an extended carboxylic-acid-functionalized PTPA (PTPA-COOH) were synthesized as ER-active materials by Buchwald–Hartwig cross-coupling. The structural, morphological, electrical, microstructural, and surface properties were investigated in detail before the ER performance was determined. Dispersions of the CMPs were prepared in silicon oil and examined under different *E* values by measuring shear stress, shear viscosity, and moduli values. PTPA-COOH dispersion with 10 wt % concentration exhibited excellent dispersion stability of 99% and enhanced ER performance, including high shear stress (static yield stress of 370 Pa at 3.5 kV/mm), repeatable and reversible electric field response, and obvious dielectric loss peak (relaxation time of 0.01 s). This class of functionalized 3D analogs of PAni shows significant promise as ER-active materials for applications in smart fluids.

## 1 Introduction

Smart or stimuli-responsive materials possess the ability to dynamically adjust or respond to various external physical or chemical triggers (such as mechanical stress, pressure, temperature, electric or magnetic fields, light, ultrasound, chemicals, pH, and ionic strength) in a controlled manner, yielding a useful outcome.^1−4^ Owing to their responses to such stimuli in terms of changing their shapes, molecular assemblies, and mechanical properties, smart materials have gained considerable attention in various applications such as sensing,^5^ biomedical devices,^6^ tissue engineering and drug delivery systems,^7^ vibration control devices, and electronics.^8^

Among stimuli-responsive materials, electrorheological (ER) fluids are categorized as smart fluids and consist of electrically polarizable particles, generally 0.1–100 μm in size, dispersed within an insulating carrier fluid such as silicone oil (SO) at volume fractions of 5–50%.^1,9^ The rheological properties of the ER fluids can be tuned by applying an external electric field (*E*). The dispersed particles are randomly dispersed in an insulating carrier fluid, and the ER fluid generally demonstrates characteristics similar to those of a Newtonian fluid when no electric field is applied. When subjected to the applied field, the differing dielectric properties between the dispersed particles and the carrier medium lead to the formation of a dipole moment, resulting in particle polarization.^10^ This induced polarization leads the dispersed particles to align and aggregate in fibrillar chains or columnar structures along the direction of the electric field between the electrodes, resulting in significant shear viscosity (or stress) and an elastic modulus increase in milliseconds; such changes in physical properties are reversible. Above a threshold point of the applied field, ER fluids exhibit solid-like behavior. With the ability of rapid and reversible transition between liquid and solid-like states under an electric field, ER fluids have found potential applications in various fields, including as microvalves, tactile displays, microfluidics systems, shock absorbers, and actuators.^11^

Developing ER fluids with the desired high performance poses a challenging task, especially when considering the wide range of variables such as type, shape, size, volume fraction, conductivity, dielectric property, surface properties of the dispersed particles, dispersing medium, temperature, externally applied electric field strength, and colloidal stability of the dispersion.^9,12^ However, considerable efforts have been made to overcome some of the shortcomings of the ER fluids, such as sedimentation,^13−15^ moisture sensitivity,^16^ high density,^15,17^ low interfacial polarization,^17−19^ thermal instability,^16,20^ and poor achieved yield stress.^16,19^ A wide range of particles has been investigated as ER-active materials to date, including silica, alumina, cellulose, starch, clay, mesoporous inorganic materials, graphene oxide, semiconducting materials, ionic crystalline materials, ionic liquids, core–shell systems,^21−24^ and inorganic/conducting polymer composites and nanocomposites.^25^ Particularly, designing hollow or porous structures in the mentioned ER active materials reduces the density of the dispersed phase and increases the surface area, thereby enhancing the dispersion stability, interfacial polarizability, and ER performance. Among the ER-active materials, conjugated semiconducting polymers have the advantages of low density, nonabrasiveness, acceptable dispersion stability, and adjustable electrical conductivity.^26^

Particularly, polyaniline (PAni)-based materials have received considerable attention as inherent polarizable anhydrous ER-active materials, with typical performance in the yield stress range of 15 Pa to 2 kPa. However, PAni can only be used as an ER-active material after carefully adjusting its conductivity by dedoping to prevent the current leakage problem to an appropriate range.^9^

Carbonaceous materials with tunable pore sizes and hierarchical porous structures have also been shown to provide specific functions and higher performance as ER-active materials.^27^ Carbonaceous particles with mesoporous and microporous pores were prepared by carbonization of template-assistant starch/silica particles at different temperatures for use as ER-active materials. The resultant mesoporous carbon-based ER fluid was reported to exhibit attractive ER properties due to its hierarchical structures.^27^ In another study, porous Ti-based metal–organic framework (MOF-Ti)@SiO_2_ core–shell nanoparticles were prepared. It was reported that the SiO_2_ shell thickness reduced the leakage current density of the nanoparticles, and enhanced interfacial polarization, while the internal pores of MOF-Ti further enhanced dispersion stability.^28^ A similar impact of the shell was also reported for PAni-coated MOF-Ti core/shell nanoparticles.^23^

Conjugated microporous polymers have garnered considerable interest for diverse application areas such as gas^29^ and dye^30^ adsorption, catalysis,^29,31^ electrochemical energy storage,^32^ sensing,^33^ and light harvesting^34^ owing to their nanopores, large surface areas, high chemical and thermal stability, reduced density, reversible redox properties, and structural modularity.^35,36^ With these attractive properties, CMPs have the potential to be suitable candidates for use in ER fluids. To the best of our knowledge, using CMPs as an ER-active material has rarely been reported in the literature: recently, a microporous covalent triazine-based polymer (MCTP) with low density and electrical conductivity within the optimal conductivity range^14^ of 10^–9^–10^–5^ S cm^–1^ was synthesized using an inexpensive precursor and used as an ER-active material.^10^ In another recent study, the above-mentioned MCTP was used to fabricate polymer–inorganic composite particles by combining nanosized Fe_3_O_4_ particles on the polymer microspheres via a chemical co-precipitation method. Results indicated that the ER properties of MCTP-Fe_3_O_4_-based ER fluids improved compared with bare MCTP.^37^ With the aid of the adjustable structure and chemical functionality of CMPs, the effects of the pore size and pore size distributions of the polymers and the amount and type of the incorporated heteroatoms in the polymer chain on ER activity can be investigated with the aim of obtaining high-performance ER fluids. Additionally, there is still significant room for improvement in the polarizability of CMPs, and thus for expanding investigations into the underexplored effects of functionalization on the ER response.

In this study, we therefore aim to explore the use of 3D equivalents of the well-known conducting polymer PAni as ER fluids. The goal of our study was not to outcompete PAni, but to provide insights into how the CMPs and functionalized CMP can improve dispersion stability and ER performance. In addition to exploring the effect and influence of the 3D structure of polytriphenylamine (PTPA), an extended carboxylic-acid-functionalized PTPA (PTPA-COOH) was synthesized by Buchwald–Hartwig coupling. The latter was prepared to enable exploration of the effect of polarizable functional groups within the CMP framework. After a detailed characterization of these materials, we investigate the effects of material density, conductivity, viscoelastic properties, and polymer loading on the properties, and field responses of the formulated ER fluids. From these investigations, we aim to produce optimized formulations, thus providing opportunities for the development of ER fluids with superior properties, performance, and applications.

## 2 Materials and Methods

### 2.1 Materials

Silicone oil (SO, polydimethylsiloxane with a viscosity of 1.0 Pa s and density of 0.967 g mL^–1^) was obtained from Sigma-Aldrich and dried at 80 °C under 25 mbar for 24 h in a vacuum oven. Once dried, the oil was used as a dispersing medium to prepare ER fluids. All other reagents and solvents were purchased from Sigma-Aldrich and used as received unless otherwise stated.

### 2.2 Synthesis of ER-Active CMPs

#### 2.2.1 Synthesis of PTPA

A Schlenk tube was charged with tris(4-bromophenyl)amine (2.5 mmol, 1205 mg), *p*-phenylenediamine (7.5 mmol, 811.05 mg), Pd(dba)_2_ (dba: dibenzylideneacetone, 129.75 mg), 2-dicyclohexylphosphino-2′,4′,6′-triisopropylbiphenyl (XPhos, 0.45 mmol, 215 mg), and sodium *tert*-butoxide (NaO*t*Bu, 1441.5 mg, 15 mmol). Anhydrous toluene (150 mL) was added, and the reaction mixture was heated to 115 °C under constant stirring for 48 h. The reaction mixture was cooled to room temperature, and the insoluble precipitated polymer was removed by centrifugation and washed with chloroform, methanol, and boiling water (200 mL each) to remove any unreacted monomers or catalyst residues. The polymer was further purified by Soxhlet extraction using chloroform, methanol, and water (24 h per solvent). The product, PTPA, was dried in a vacuum oven at 60 °C for 48 h and isolated as a blackish powder.^38^

#### 2.2.2 Synthesis of Extended −COOH Functionalized PTPA (PTPA-COOH)

A Schlenk tube was charged with tris(4-bromophenyl)amine (2.5 mmol, 1205 mg, 1 equiv), *p*-phenylenediamine (15 mmol, 1622.1 mg, 6 equiv), 2,5 dibromobenzoic acid (7.5 mmol, 2099.325 mg, 3 equiv), Pd(dba)_2_ (129.75 mg), XPhos (0.45 mmol, 215 mg), and NaO*t*Bu (3603.75 mg, 37.5 mmol). Please note that an excess base (NaO*t*Bu) was added to ensure that the acidic 2,5-dibromobenzoic acid would not interrupt the catalytic cycle (see Scheme S1 for the Buchwald–Hartwig catalytic cycle, highlighting the important role of the base). Anhydrous toluene (150 mL) was added, and the reaction mixture was heated to 115 °C under a N_2_ atmosphere and constant stirring for 48 h. The reaction was cooled to room temperature, and the insoluble precipitated polymer was removed by centrifugation and washed with chloroform, methanol, and boiling water (200 mL each) to remove any unreacted monomers or catalyst residues. The polymer was further purified by exhaustive Soxhlet extraction using chloroform, methanol, and water, respectively (24 h per solvent). The product was dried in a vacuum oven at 60 °C for 48 h and isolated as a blackish powder.

### 2.3 Characterization

A PerkinElmer Spectrum Two Fourier transform infrared (FTIR) spectrometer, equipped with a universal single reflection diamond attenuated total reflectance (UATR) attachment, was utilized to record the FTIR spectra of the powder samples. The spectra were recorded in the wavenumber range of 4000–400 cm^–1^.

Ultraviolet–visible–Near Infrared (UV–vis–NIR) spectra of the materials blended with barium sulfate (BaSO_4_) were acquired by utilizing a Shimadzu UV2600 spectrophotometer, fitted with an ISR2600Plus integrating sphere attachment. The spectra were recorded in the wavelength range of 220–1400 nm.

To obtain information regarding polymer thermal stability, thermogravimetric analysis (TGA) was conducted using a NETZSCH STA 449 F1 Jupiter in accordance with criteria published in ISO 11358-1:2022.^39^ Approximately 5–10 mg of sample was placed in an alumina crucible (Al_2_O_3_) and heated in an atmosphere of nitrogen (N_2_) at a rate of 5 °C min^–1^ within the temperature range of 100–1000 °C. The pure gas flow rate was 50 mL min^–1^. The protective gas flow rate was 20 mL min^–1^. To eliminate any adsorbed moisture content, an isothermal dwell was maintained at 100 °C for 30 min before data collection, and the initial mass change was corrected accordingly. Analysis using the same parameters and empty crucibles was performed to create accurate correction data sets before the experiment was performed on the samples. The extrapolated initial thermal onset temperature (*T*_0_) calculated by the procedure detailed in ISO is also reported for each material.

For the surface area measurements, samples were dried on a Schlenk line at 150 °C under vacuum conditions for 24 h. Gas sorption measurements were carried out by utilizing a Quantachrome Autosorb-1MP instrument. Before sorption measurements were conducted, the samples underwent a three-step degassing process under a high vacuum. Initially, the sample was gradually heated at a rate of 1 °C/min to 50 °C and maintained at that temperature for 45 min. Subsequently, the temperature was raised to 100 °C at a rate of 2 °C/min, with the sample held at this temperature for 100 min. Finally, the sample was heated to 180 °C at a rate of 2 °C/min and maintained at this temperature for 600 min. Nitrogen adsorption–desorption measurements were performed at 77.4 K. Specific surface areas were determined utilizing the Brunauer–Emmett–Teller (BET) model applied to the adsorption or desorption branches of the nitrogen isotherms at 77.4 K. Calculations were carried out using the QuadraWin 5.05 software package. Additionally, pore sizes were analyzed using commercial nonlocal density functional theory (NLDFT) incorporated in the QuadraWin 5.05 software package.

The morphology of the obtained CMPs was investigated after the grinding process using scanning electron microscopy (SEM, Hitachi SU5000, Japan) with an accelerating voltage of 20 kV. Prior to imaging, samples were coated with a thin layer of gold using a sputter coater (Leica EM ACE200, Germany), employing a gold target of 99.99% purity.

Contact angle measurements were conducted utilizing a Kruss Drop Shape Analyzer DSA 100 instrument, with data analyzed using the ADVANCE software (Kruss, Version 1.10.0). Samples in pellet form with a diameter of 1.3 cm were employed. A 10 μL droplet of deionized water or SO was deposited onto the surface, and the change in contact angle was monitored using a camera over a period of 300 s (1 measurement per second).

Electrical conductivity measurements were conducted at 25 °C using the four-probe technique on a compressed disc-shaped pellet with specific dimensions (13.00 mm in diameter and approximately 0.50 mm in height, Entek Electronic, FPP 470-A, Turkey). The average electrical conductivity values were derived from measurements taken at a minimum of five distinct locations on the pellet. Additionally, the prepared pellets were utilized to determine the apparent density of the samples through the calculation of their volume and the measured mass of each pellet.

### 2.4 Preparation of ER Fluids

Typically, particle sizes in the range 0.1–100 μm are acceptable for the dispersed phase of ER fluids.^1^ To obtain fine-grade polymer powders for further use, PTPA and PTPA-COOH particles were ground using a ball mill at 20 Hz for 10 min (Retsch, MM400, Germany), and the particle size distributions of the CMPs were measured by laser diffraction analyzer (Malvern, Mastersizer 2000, U.K.); the *d*(0.5) value was determined as 15.2 and 13.5 μm for PTPA and PTPA-COOH, respectively. The ER dispersions were prepared by dispersing PTPA and PTPA-COOH at mass percent concentration series of 2.5–10.0 wt % in SO with 2.5% increments. The dispersions were mixed mechanically and then subsequently uniformly mixed using a probe sonicator (500 W, 20 kHz) at 20% amplitude for 30 s with an on/off pulse cycle of 3 s to avoid overheating (Sonics, Vibracell, USA) before being employed in rheological, dielectric, and dispersion stability measurements.

### 2.5 Dielectric Measurements of the ER Fluids

The dielectric spectra of the PTPA/SO and PTPA-COOH/SO dispersions (10.0 wt %) were measured using an impedance analyzer (Agilent E4980A precision LCR meter equipped with a 16452A model liquid test fixture, Japan) to determine their dielectric spectra within the frequency range from 20 Hz to 1 MHz. The bias potential was 1.0 V.

### 2.6 Dispersion Stability Measurements

The resistance of dispersed particles to sedimentation in the ER fluids was assessed by measuring the separation height between particle-rich and clear oil-rich phases over time at room temperature (25 °C) using a digital caliper. The antisedimentation ratios (%) of the dispersions were determined by calculating the percentage of the height occupied by the dispersed particle-rich portion relative to the total height of the dispersion.

### 2.7 ER Measurements on the Prepared Fluids

The rheological measurements were conducted at 25 °C using a torque rheometer (Thermo-Haake RS600 Rheometer, Germany) equipped with 35 mm parallel plates spaced 1.0 mm apart.

The DC electric field generator (HCL 14, FuG Electronik, Germany) was linked to the rheometer for the application of *E*. Prior to each measurement, the dispersions underwent preshearing at a rate of 60 s^–1^ for 60 s, followed by a 60 s equilibration period without shear under the desired *E* values.

The flow curves were determined by measuring shear stresses (τ) and viscosities (η) as functions of shear rates (γ̇) at various *E* values in the controlled shear rate (CSR) mode. Additionally, to determine the static yield stress, the measurements were performed in a controlled shear stress (CSS) mode, in which a linearly increasing shear stress was applied to the dispersions under *E*. The static yield stress (τ_sy_) values were obtained from the curves of γ̇ vs τ in CSS mode as the stress values where a rapid jump in the γ̇ was observed, and the measured shear rate became significant.^40^ The on–off *E* responses were conducted by measuring the τ values over time at a fixed shear rate of 1.0 s^–1^ by switching the *E* alternately applied and removed at a fixed time interval of 30 s for the 10 wt % dispersions. The speed of the on–off *E* response and the reversibility of the phase change were monitored under different *E* values by increments of 0.5 kV/mm as well as under a fixed *E* value of 3.5 kV/mm. Dynamical oscillatory rheological measurements were conducted to elucidate the viscoelastic properties of the PTPA-COOH dispersion (10 wt %). First, to identify the linear viscoelastic regions (LVER) across a range of electric field strengths (from 0 to 3 kV mm^–1^ in 1 kV mm^–1^ increments), elastic and viscous modulus values were measured over shear stress at a fixed frequency of 1 Hz. The LVER indicates the range in which the oscillatory test can be carried out without destroying the microstructure of dispersion. The LVER was determined by observing the limit of the plateau region of elastic modulus (*G*′) versus the stress curve after where the *G*′ became stress-dependent. Second, the viscoelastic moduli were measured over a frequency range of 0.1–100 Hz at constant stress values in the predetermined LVER at various *E* values.

## 3 Results and Discussion

**Scheme 1:**
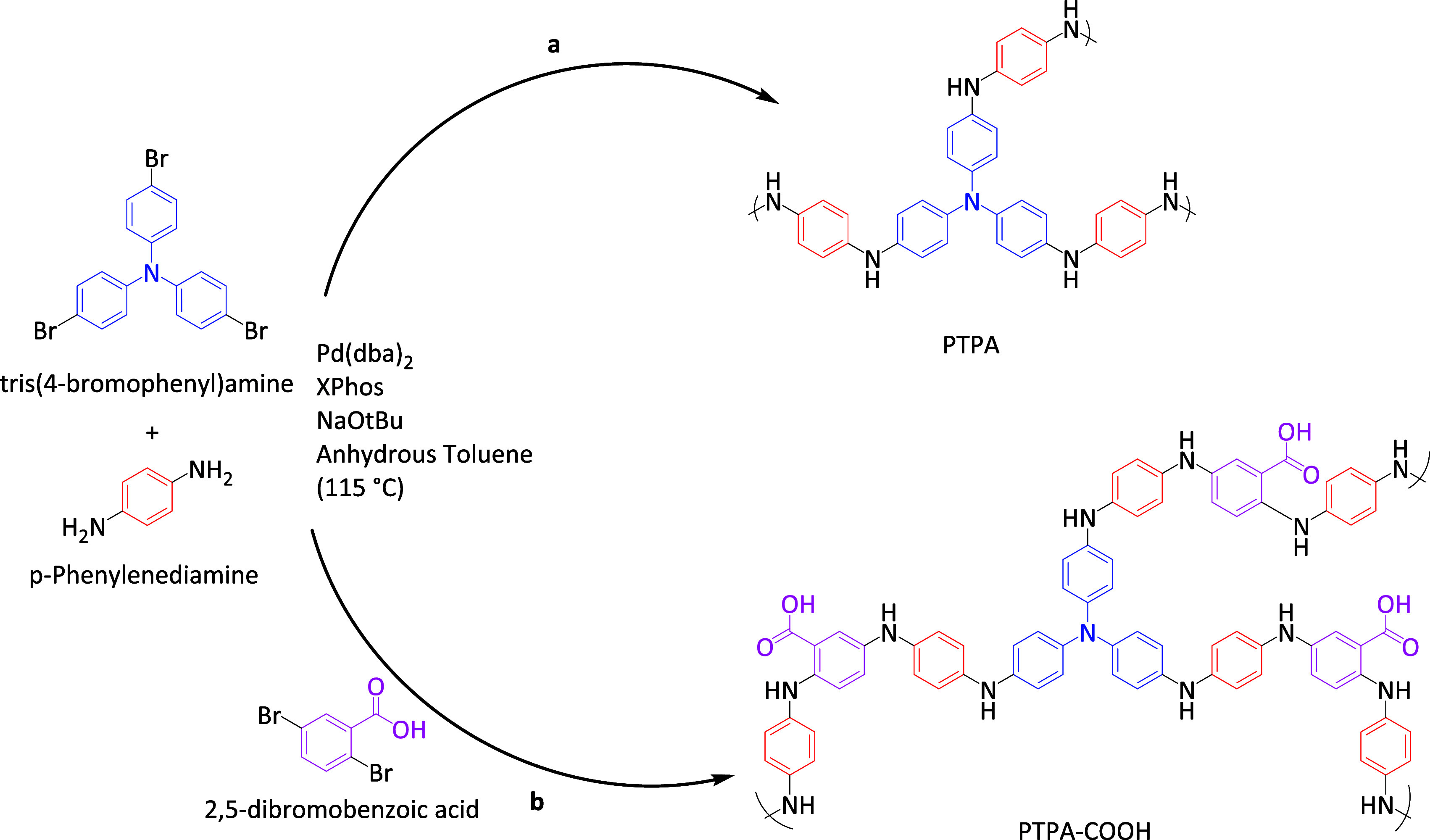
Synthetic Route for the Formation of PTPA (a) and PTPA-COOH (b) Networks

Two materials were identified as candidates to explore their ER properties: the first is a “standard” CMP, PTPA, which has been investigated for a range of applications related to CO_2_ capture and conversion and wastewater treatment.^30,38^ This material, based on a tris(4-bromophenyl)amine core and *p*-phenylenediamine linker, will, on reaction in the presence of a Pd catalyst, undergo cross-coupling (a so-called Buchwald–Hartwig cross-coupling reaction) to yield a highly cross-linked 3D version of the well-known conducting polymer, PAni (Scheme 1a). PAni, as outlined above, has been extensively used in investigations for potential ER responses;^41^ PTPA is, therefore, a logical next step for exploration for further ER applications. To further explore this motif, we synthesized a PTPA-based system with additional functionality using a carboxylic acid-functionalized comonomer, 2,5-dibromobenzoic acid (Scheme 1b). The aim was to incorporate a highly polarizable functional group into the backbone and framework of this 3D PAni analog.

### 3.1 Characterization

**Figure 1:**
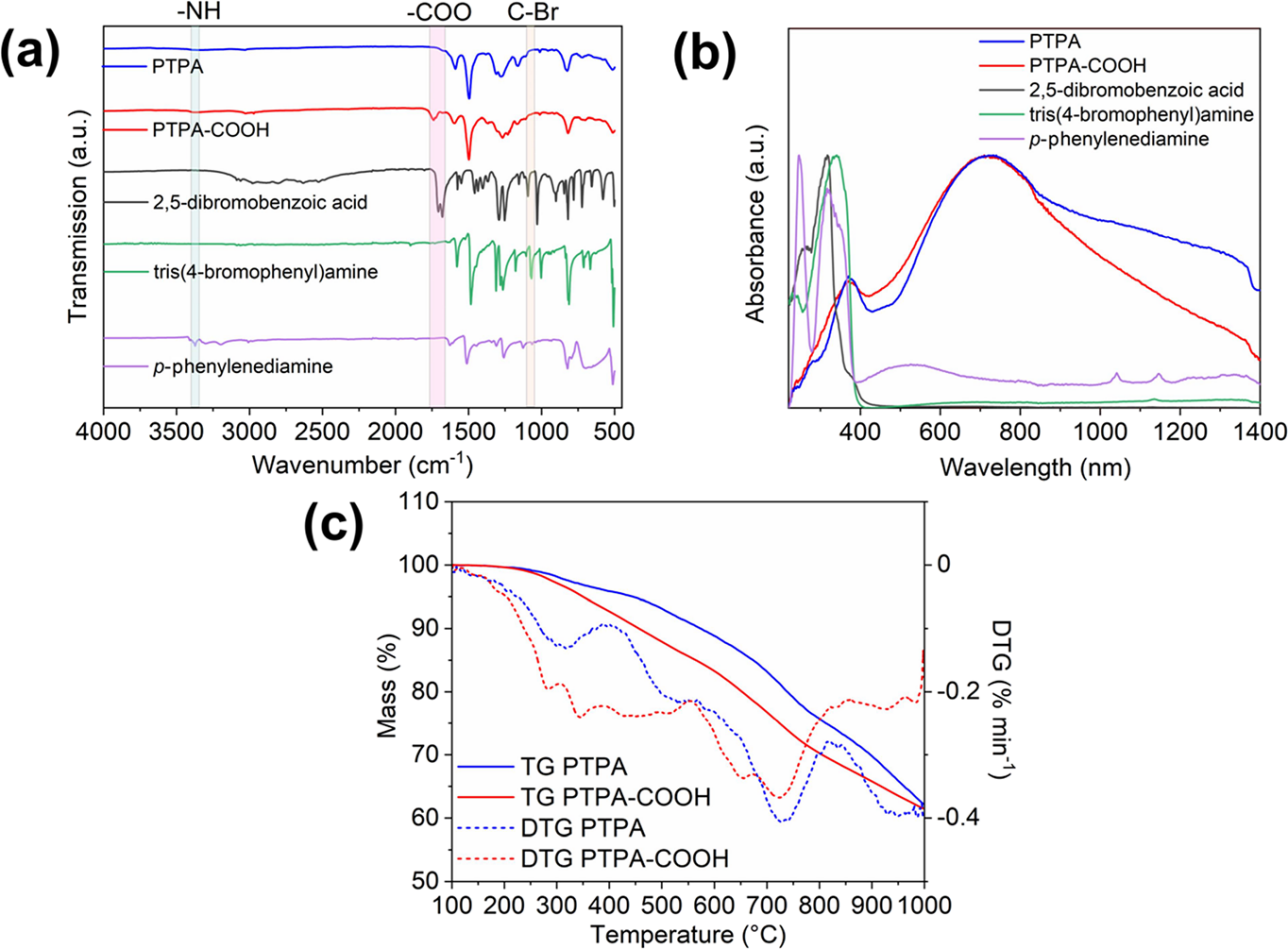
FTIR spectra (a) and solid-state UV–vis–NIR spectra (b) of starting materials tris(4-bromophenyl)amine, *p*-phenylenediamine, 2,5-dibromobenzoic acid, PTPA and PTPA-COOH, and TGA curves (c) of PTPA and PTPA-COOH.

FTIR spectroscopy investigations confirmed reaction completion by the vanishing or strong attenuation of the bands relating to aromatic C–Br stretching (1068 cm^–1^) from tris(4-bromophenylamine) and -NH_2_ stretching (3371 cm^–1^) from the diamine, Figure 1a.^38,42^ The peak associated with the carboxyl group stretching (1737 cm^–1^) in the extended carboxylic-acid-functionalized PTPA (PTPA-COOH) was comparable with the benzoic acid bands in the starting 2,5-dibromobenzoic acid, indicating no change in the desired carboxyl functionality.

For both materials, the solid-state UV–vis–NIR spectra showed a broad peak at ∼710 nm and a weak peak at ∼370 nm. These peaks were ascribed to the π–π* transition of quinoid and benzenoid rings, respectively (Figure 1b),^38,43^ as typically found for PAni.

The thermal decomposition behavior of both PTPA and PTPA-COOH under a N_2_ atmosphere is presented in Figure 1c. The onset decomposition temperature (*T*_0_) was determined as 269 °C for PTPA and 242 °C for PTPA-COOH. The derivative thermogravimetric (DTG) analysis, which quantifies the rate of mass loss, revealed that PTPA-COOH undergoes initial thermal decomposition in two distinct peaks within the 280–343 °C range. The lower onset temperature and increased decomposition rate of PTPA-COOH suggest the presence of thermally labile, oxygenated functional groups such as carboxylic acid (−COOH) groups. This behavior aligns with literature attributing thermal degradation in this range to the decarboxylation of carboxyl groups.^44−47^ In comparison, PTPA displayed a simpler decomposition profile in the same range with a smaller mass loss, supporting the absence of these surface functionalities. Between 280 and 400 °C, PTPA exhibited a 2.8% mass loss, whereas PTPA-COOH showed a higher mass loss of 5.2%, reflecting its greater decomposition. At 950 °C, the mass loss of both materials converged (∼35%), indicating reduced specific decomposition processes at elevated temperatures. The char yields of these materials are typical values to be expected for such highly cross-linked CMP systems, as again shown in a recently published study.^48^

**Figure 2:**
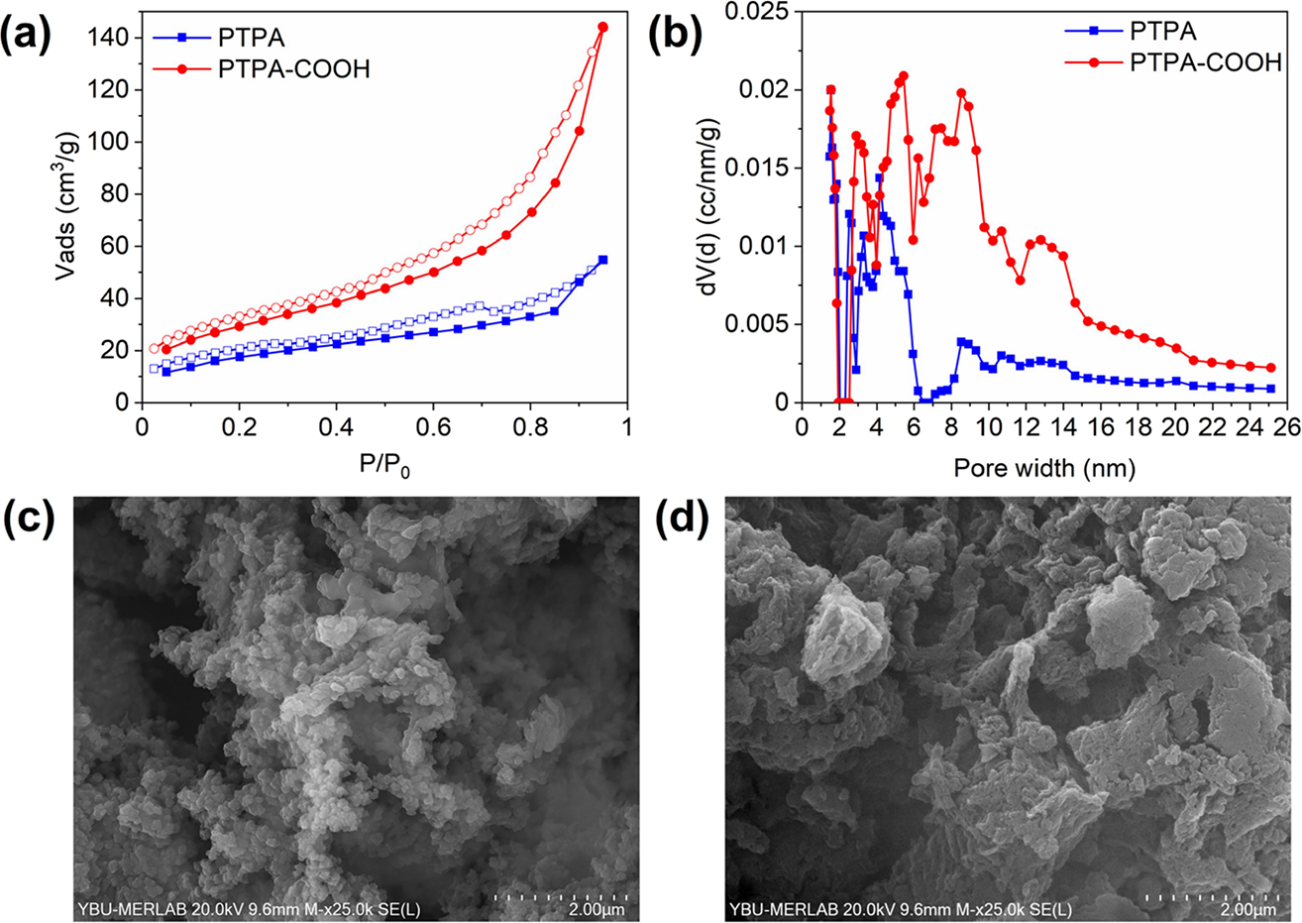
N_2_ isotherms (a) and PSDs (b) of CMPs and SEM image of (c) PTPA (d) and PTPA-COOH.

N_2_ adsorption–desorption measurements indicated a type IV isotherm for both materials (Figure 2a), which is indicative of mesoporous materials.^49^ Pore size distributions (PSDs) were studied to show the pore sizes of materials and their contribution to the overall surface areas. Both polymers exhibited broad PSDs as shown in Figure 2b. The specific surface area (*S*_BET_) of the PTPA and PTPA-COOH were determined as 66 and 108 m^2^g^–1^, with pore volumes of 0.080 and 0.214 cm^3^g^–1^, respectively.

SEM images (Figure 2c) revealed the typical fused nanoparticle morphology, as previously found for PTPA. PTPA-COOH (Figure 2d) exhibits more fused structures with fewer discernible particles.

The ability for the electron distribution to be distorted in the polymeric structure promotes the ER effect when subjected to an external electric field.^10^ Hence, the electrical properties (i.e., electrical conductivity and dielectric constant) have an essential effect on the ER performance of the ER fluids. The conductivity of PTPA and PTPA-COOH was 3.1 × 10^–6^ and 1.6 × 10^–6^ S cm^–1^, respectively. The conductivity of −COOH functionalized PTPA was slightly higher than that of PTPA. According to the conductivity results, the conductivities of PTPA and PTPA-COOH were in the conductivity range of semiconductors and, thus, were also in the recommended conductivity range for ER-active materials. If the electrical conductivity exceeds the optimal range for the ER application, then there is a risk of electrical short circuits when applying an electric field (*E*) to an ER fluid. Therefore, it is often necessary to dedope most semiconducting polymer-based particles using appropriate methods to achieve the desired conductivity.^41^ In addition to the influence on the ER performance, the conductivity of the dispersed particles also plays a key role in affecting the current density and response time of the entire ER fluid.^50^ It is noteworthy that no dedoping process was necessary for the CMPs prepared in this study; the current intensity did not exceed 1.0 × 10^–3^ mA during the ER measurements of the dispersions within the examined *E* range.

### 3.2 Dielectric Properties of the CMP-Based ER Fluids

When an external electric field is applied to an ER fluid, interfacial polarization occurs between the dispersed particles and the insulating carrier fluid and contributes to the ER effect rather than other types of polarizations, i.e., electronic, and atomic polarizations.^51^ Therefore, dielectric spectra of ER fluids offer valuable insights into the polarization mechanism, polarizability, and polarization relaxation time of the ER fluids when examining their electrical polarization properties and interpreting the flow characteristics of the ER dispersion under the electric field.^52^ In general, a greater polarizability of the dispersed particles yields stronger electrostatic interactions and thus enhances their ER performance. On the other hand, the response time of the dispersed particles to the electric field and the formation of stable and robust fibrillar-like structures within the insulating carrier fluid usually depend on the relaxation frequency of the ER fluid. A rapid relaxation time occurs when the relaxation frequency is high. It was reported that neither too high nor too low of the polarization rate was beneficial for achieving a strong ER effect experimentally,^51^ and the relaxation frequency of the ER fluid was generally recommended to be in the frequency range of 10^2^–10^5^ Hz (in addition to possessing a strong interfacial polarizability).^53,54^ Thus, if the relaxation time is excessively low (below 1.59 × 10^–6^ s), the reconstruction of the chain-like structures during shear deformation might be hindered. Conversely, when the response time is excessively high (above 1.59 × 10^–3^ s), the repulsion between particles becomes dominant, leading to a decrease in the stability of the chain-like structures and a consequent negative impact on performance as ER fluid.^51,55,56^

**Figure 3:**
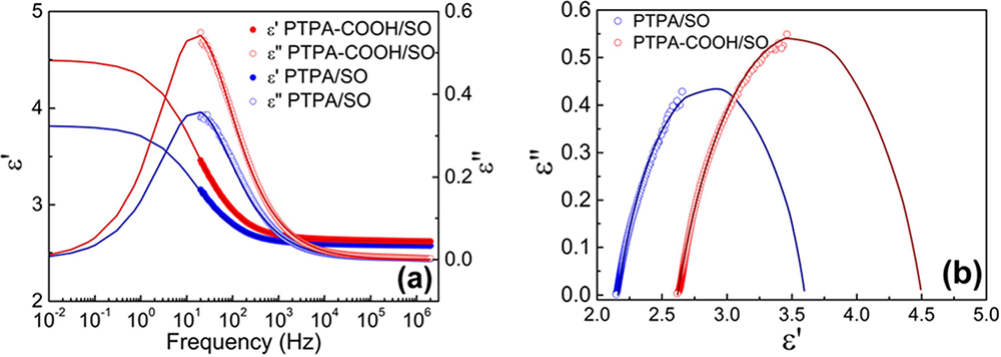
Dielectric spectra (a) and Cole–Cole plot (b) of the PTPA/SO and PTPA-COOH/SO dispersions (10.0 wt %).

The spectra of the dielectric constant (ε′) and dielectric loss (ε″) of the 10 wt % PTPA/SO and PTPA-COOH/SO dispersions are shown in Figure 3a. The experimental complex dielectric permittivity of the ER dispersions, ε*(ω), was fitted by the Cole–Cole equation (eq 1) for a single relaxation process:^57,58^
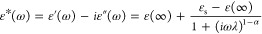
1where ω is the angular frequency (ω = 2π*f*, *f* is the frequency applied), λ is the relaxation time, and λ = 1/2π*f*_max_, wherein *f*_max_ denotes the frequency associated with the dielectric loss peak, α is an empirical parameter that characterizes the broadness of the loss peak or distribution of relaxation times, and ε_s_ and ε(∞) represent the minimum value of the dielectric constant at low frequencies and the dielectric constant approaching limit at high frequencies, respectively.

The fitted curves provide dielectric spectra of the dispersions at low and high frequencies, facilitating the calculation of the dielectric parameters; the obtained relevant dielectric parameters are presented in Table 1. The variation in ε′ values between low and high frequencies approaching the limit (Δε = ε_s_ – ε(∞)) serves as an indicator of the dielectric relaxation strength and the achieved polarizability of the material. λ is associated with the response time of the material to the electric field. The complex plane (Cole–Cole) plot, where ε″ is plotted against ε′, can be utilized for the analysis of dielectric relaxation: if a semicircle is formed when the complex plane is drawn, the dielectric relaxation demonstrates a single relaxation time, i.e., Debye-type relaxation.^59^ When there is a deviation from a semicircular shape or the appearance of asymmetric curves in the complex plane plot, variations in relaxation times or the existence of multiple relaxation times can be suggested. According to the Cole–Cole plot, both dispersions showed a single relaxation caused by the interfacial polarization of the particles (Figure 3b).

**Table 1:** Dielectric Parameters of PTPA/SO and PTPA-COOH/SO Dispersions (10.0 wt %)

dispersion	ε_s_	ε(∞)	Δε = ε_s_–ε(∞)	*f*_max_ (Hz)	λ (s)	α
PTPA/SO	3.70	2.15	1.55	10	0.016	0.32
PTPA-COOH/SO	4.50	2.62	1.88	16	0.010	0.33

The relaxation frequencies of both dispersions were not found in the expected range but were located in a much lower frequency range. Although reasonable fast relaxation contributes to a high ER effect, it is not as critical as having high dielectric relaxation strength which may primarily influence the electrostatic attraction between particles.^60^ However, the relatively shorter relaxation times of the dispersions of PTPA/SO and PTPA-COOH/SO, both of which were within the interfacial polarization range, suggested that upon the application of an electric field, the dispersed particles in the dispersions could swiftly reorganize their internal chain-like structure. Furthermore, the dielectric properties are not the only factors affecting the ER performance and an appropriate level of conductivity is also required. The PTPA-COOH/SO dispersion displayed enhanced dielectric relaxation strength and shorter relaxation time compared with the PTPA/SO dispersion, which suggested better interfacial polarization ability owing to the presence of more polarizable −COOH groups in the structure. This observation is also consistent with the ER performance discussed in the following section. Furthermore, the increased surface area and hierarchical porous structure of PTPA-COOH facilitated an easy and efficient transfer of polarization, improving the surface polarization response of the particles. A similar relatively higher dielectric relaxation strength was reported for mesoporous carbonaceous structures compared with microporous carbon in the literature.^27^ In another study, a slightly improved achievable polarizability with shorter relaxation time was reported for PAni when using the larger dopant anion indole-2-carboxylic acid during the PAni nanofiber synthesis process (instead of the commonly used hydrochloric acid).^61^

### 3.3 Dispersion Stability of the CMP-Based ER Fluids

**Figure 4:**
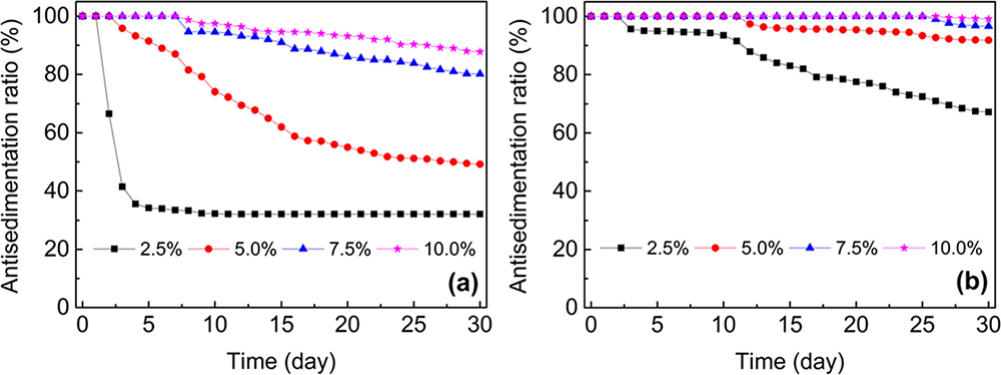
Change in the antisedimentation ratio of (a) PTPA/SO and (b) PTPA-COOH/SO with time for 2.5–10.0 wt % particle concentration.

ER fluids are generally composed of dispersed ER-active particles and an insulating carrier fluid. Thus, the stability of the dispersion should be maintained, and no sedimentation is desired during application and long-term usage. A lower density mismatch between the dispersed and dispersing phases and good wettability of the particles in the carrier fluid can result in enhanced dispersion stability. For the dispersion stability tests of our materials, the dispersions were allowed to settle undisturbed for 30 days, and dispersion stabilities were determined by measuring the antisedimentation ratio as a function of time at 25 °C for 2.5–10.0 wt % concentrations. The antisedimentation ratio is a measure of dispersion stability and is determined by calculating the percentage of the height occupied by the dispersed particle-rich portion relative to the total height of the dispersion in the time period examined. The antisedimentation ratio reached an equilibrium value faster at lower concentrations for both dispersions (Figure 4). The reason can be attributed to the increased particle–particle and particle–carrier fluid interactions and dispersion viscosity with increasing dispersion concentration, and the predominance of the gravitational forces for lower particle concentrations.^62^ Similar concentration-dependent dispersion stability tendencies were reported for particulate PAni, PAni nanoparticles, and nanofibrous PAni in the literature.^63,64^ In a study by Gercek et al., the dispersion stability of PAni derivative dispersions in SO, including poly(*o*-toluidine), poly(*N*-methyl aniline), poly(*N*-ethyl aniline), and poly(2-ethyl aniline) particles with similar morphology and particle sizes, were investigated; similar or poorer antisedimentation ratios (around 54%) for 5 wt % particle concentration were reported after 30 days.^65^ In another study, the effect of nonionic surfactant addition on the dispersion stability of poly(3-aminophenyl boronic acid) and poly(thiophene-3-boronic acid) dispersions in SO was investigated by Ozkan et al., and 77 and 100% antisedimentation ratios were reported for poly(thiophene-3-boronic acid) and poly(3-aminophenyl boronic acid), respectively, after 30 days. However, the dispersion stabilities of the bare dispersions without surfactant were not investigated nor reported to evaluate whether the main contribution to the enhanced dispersion stability was the addition of surfactant or the polymer structure.^66^

Yin et al. reported better dispersion stability for anisotropic fiber-like PAni (87%, after standing 500 h) relative to granular PAni (43%, after standing 500 h) (both at 10 wt % concentration), owing to possessing a decreased sedimentation velocity and larger drag index, in particular, for high particle concentrations.^63^ Our CMP-based ER dispersions displayed improved dispersion stability even without the addition of any stabilizing additives or having an anisotropic structure compared with the PAni-based materials reported in the literature. The highest dispersion stabilities of 87 and 99% for PTPA/SO and PTPA-COOH/SO ER dispersions, respectively, were observed at 10 wt % particle concentration after 30 days. The PTPA-COOH/SO dispersion showed enhanced dispersion stability compared with PTPA/SO, even though both have similar density values (the apparent density of the PTPA and COOH-PTPA is 0.88 ± 0.02 and 0.91 ± 0.01 g cm^–3^, respectively). This enhancement can be attributed to the improved interfacial adhesion between the carrier fluid and PTPA-COOH, enhanced wettability, surface area, and greater hierarchical porous structure compared to those of the bare PTPA particles. In a previous study, carbonaceous materials with mesoporous structures demonstrated better dispersion stability (approximate antisedimentation ratio of 90% after 30 days for 10 wt % particle concentration) than that of materials only with micropores, attributed to larger drag resistance resulting from the porosity.^27^ It is assumed that the greater hierarchical porosity present in the PTPA-COOH structure contributed to the observed high dispersion stability of the PTPA-COOH/SO samples.

Water contact angle measurements provided insight into the PTPAs’ surface and wetting characteristics (Figure S1). A drop of water was placed on the sample in the form of a pellet and measured every second for 300 s. With a contact angle of θ = 107° PTPA is classified nonwetting-hydrophobic, whereas PTPA-COOH exhibited hydrophilic wetting properties (θ = 64°), which is attributed to the presence of the additional −COOH moieties in the PTPA-COOH structure. The contact angle measurement conducted with SO as the wetting liquid yielded no values for the contact angle, as the drop of SO spread on both sample surfaces before an accurate image could be captured. These results underlined the excellent wettability of both samples with SO, despite the dispersion stability of PTPA-COOH being superior to that of PTPA.

**Figure 5:**
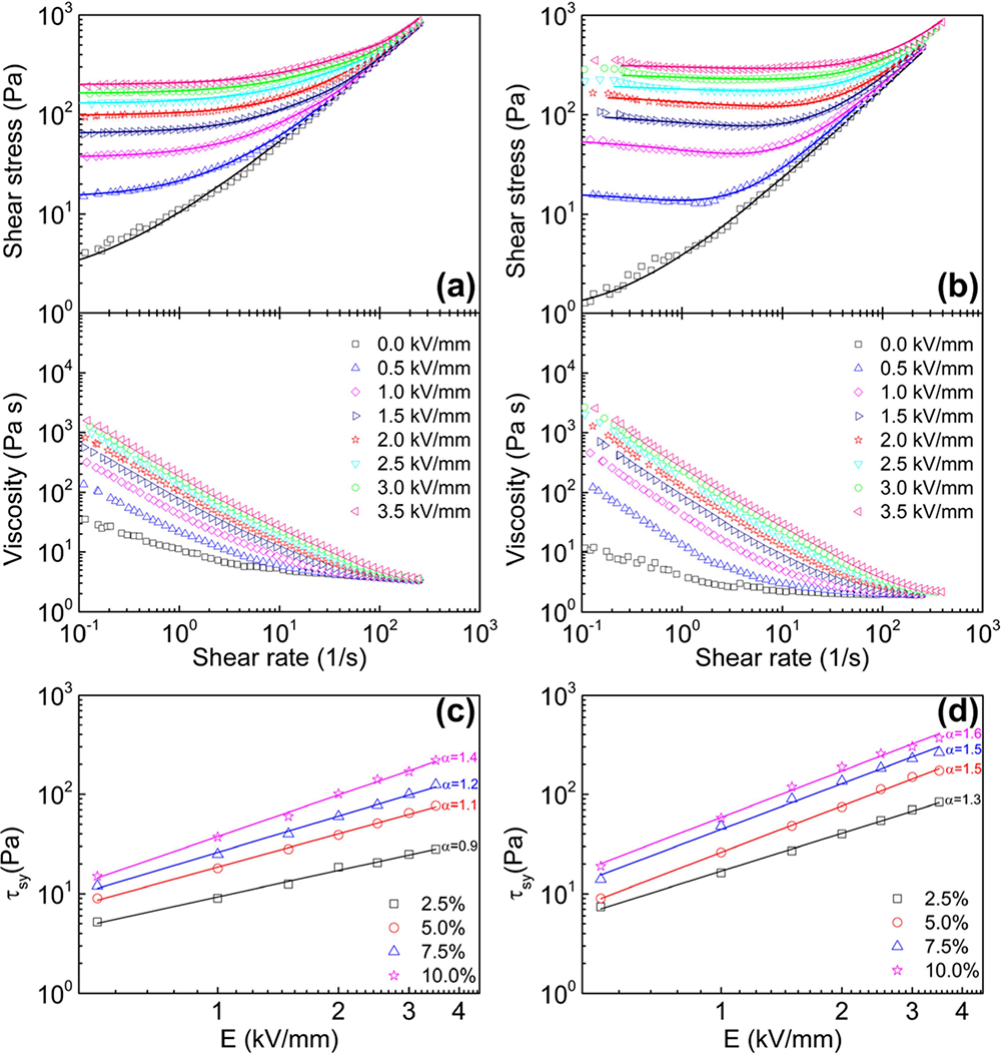
Flow curves for the PTPA/SO (a) and PTPA-COOH/SO (b) dispersions for 10 wt % (the solid lines in the shear stress–shear rate curves represent the fit of the CCJ model). The static yield stresses as a function of the electric field obtained in the CSS mode of the PTPA/SO (c) and PTPA-COOH/SO (d) dispersions at different dispersion concentrations (the corresponding index parameters were inserted).

Song et al. investigated the effect of the temperature of thermal treatment on the wettability of calcium–titanium–oxygen precipitates (CTO, composed of calcium oxalate dehydrate and titanium–oxygen precipitates) with SO. They found that the zero-field viscosity decreased while the wetting behavior of CTO decreased with the decomposition of the unconfined polar groups, such as the carboxyl group, after heat treatment, resulting in weaker particle–liquid interactions.^67^ We therefore proceeded to investigate the additional contribution of this enhancement in wetting behavior for our samples by evaluating and comparing the viscosities of the dispersions in the absence of electric fields. According to the flow curves of the dispersions measured under no electric field (Figure 5a,b), zero-field viscosity was observed as 3.4 and 1.9 Pa s at 245 s^–1^ for PTPA/SO and PTPA-COOH/SO, respectively. The enhanced dispersed particle–liquid interactions for PTPA-COOH/SO resulted in lower zero-field viscosity and greater dispersion stability, owing to the presence of free carboxylic acid groups for PTPA-COOH.

It is noteworthy that for concentrations above 10 wt % particle concentration, dispersions could not be prepared for PTPA; a paste-like appearance was formed due to the weaker particle–liquid interactions. Thus, the maximum working concentration for the CMP dispersions was chosen as 10 wt % for this study. The concentration of the dispersed phase is an important parameter that influences ER efficiency and antisettling performance. If the concentration of the dispersed phase is too high, this may lead to a lower ER efficiency, which is not beneficial for practical applications. An adequate ER effect may not be observed if the concentration is too low. In addition to affecting the rheological properties, the larger the particle concentration is, the better the antisettling performance (but which requires a fine balance to avoid the formation of nondesired high viscosity phases, such as the paste-like appearance formed in the absence of an electric field).

### 3.4 Electrorheological Properties of Our CMP-Based ER Fluids

Figure 5a,b shows the flow curves of 10 wt % PTPA/SO and PTPA-COOH/SO dispersions. In the absence of the electric field, both dispersions displayed non-Newtonian flow with shear-thinning behavior, showing relatively low yield stresses due to the formation of particle networks from interactions between dispersed particles. Upon applying an electric field, the rheological characteristics of the dispersions, such as viscosities and shear stresses, were raised significantly with *E*, demonstrating pseudoplastic behavior accompanied by yield stresses attributed to the formation of fibrillar/columnar structures. Furthermore, the shear stresses exhibited a gradual increase across the entire range of shear rates as the electric field strengths increased.

The Cho–Choi–Jhon (CCJ) model was utilized to characterize the flow behavior of the PTPA/SO and PTPA-COOH/SO dispersions, as illustrated by eq 2 provided below:^68^
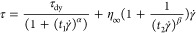
2where τ_dy_ shows the dynamic yield stress, α represents the correlation with stress (τ) reduction, *t*_1_ and *t*_2_ denote time constants, and η_∞_ the viscosity at high shear rates (γ̇), indicating viscosity under conditions where an electric field is absent. The value for β falls within the range of 0 < β ≤ 1, since dτ/dγ̇ ≥ 0. The optimal parameters in the CCJ model equation obtained from the flow curves of PTPA/SO and PTPA-COOH/SO dispersions (10 wt %) at various electric field strengths are presented in Table S1.

At low shear rates, the primary interactions among the dispersed particles are driven by electrostatic forces induced by *E*, rather than hydrodynamic interactions caused by shear flow. As the shear rate further increased, the chain-like formations of particles began to disintegrate. Above the critical shear rate, the rate at which fibrillar or columnar structures were broken down surpassed the rate at which columns were reformed by the electric field. As a result, the flow curves exhibited behavior akin to those observed in the absence of the electric field. At elevated shear rates, the fibrillar structures were disrupted into particles or particle clusters due to the prevailing influence of hydrodynamic interactions during shearing.

For the PTPA-COOH dispersion, the shear stress exhibited a slight decrease with respect to the shear rate before subsequently increasing again.^68^ This behavior suggested that the fibrillar structures formed under the electric field were relatively unstable and weaker. As a result, the reformation of these induced fibrillar structures occurred at a slower pace compared to their destruction, resulting in reformed structures that differed from those prior to shear deformation.^69−71^ However, it was noted that the PTPA-COOH/SO dispersions maintained their fibrillar structure across a broad range of shear deformations. Notably, the introduction of −COOH groups into the PTPA structure led to an improvement in their electric field flow response and resulted in improved shear stress and viscosity values with increasing *E*. Also, the shear stress of the PTPA/SO and PTPA-COOH/SO dispersions increased with increasing applied electric field without any significant leaking current density passing through the dispersion within the studied *E* range (<0.03 μA cm^–2^). Thus, both dispersions are expected to require low power consumption and ensure safety from a practical perspective.

Figure 5c,d presents the τ_sy_ values determined from the shear rate vs shear stress curves in CSS mode (Figures S2 and S3), illustrating their correlation with dispersion concentration and *E*. The τ_sy_ values represent the shear stress levels at which a rapid increase in the shear rate occurred, indicating that the measured shear rate had become notably significant. It was noted that τ_sy_ increased as particle concentration and the *E* values were increased, indicating strengthened particle–particle interactions and electrostatic forces capable of withstanding hydrodynamic forces. The increase in τ_sy_ with *E* and concentration was more profound for PTPA-COOH/SO dispersions (Figure 5d) compared with PTPA/SO dispersions (Figure 5c) owing to the synergistic effect of the existence of more polarizable functional groups in the PTPA-COOH structure and better hierarchical porous structures.

The quantitative relationship between τ_sy_ and *E* is expressed with the power-law relation of τ_sy_ ∝ *E*^α^, where α serves as the index parameter representing the slope of the fitted curve. The slope of the fitting curve for the polarization model is α = 2, while for the conduction model, it is α = 1.5.^72^ In certain instances, this correlation does not entirely match the expected index parameter of the above-mentioned models due to several unquantifiable effects of variables, such as dispersed particle size, morphology, surface properties and concentration, and dielectric properties of the ER fluid.^55^ The slope of the τ_sy_ curves for PTPA-COOH/SO was determined as 1.3, 1.5, 1.5, and 1.6 for 2.5, 5.0, 7.5, and 10.0 wt % concentration dispersions, respectively. The α value for PTPA/SO was obtained as 0.9, 1.1, 1.2, and 1.4 for the 2.5, 5.0, 7.5, and 10.0 wt % concentration dispersions, respectively. With an increasing dispersed particle concentration, the behavior approached the conduction model. It was predicted that under a DC or a low-frequency field, the larger the mismatch of the conductivity of dispersed particles and dispersing medium is, the higher the yield stress of the ER fluid. The conductivity of silicone oil is typically low, making it an effective electrical insulator. Previous studies have reported silicone oil to have a conductivity in the range of 10^–12^–10^–14^ S/cm.^73−75^ Therefore, it can be inferred that the behavior of PTPA-COOH particles in SO was determined more by the conductivity mismatch between the particles and carrier medium rather than the mismatch of dielectric constant.^76^

The dielectric spectrum interpretation was also consistent with the determined conduction model, because λ was determined as approximately 0.01 s, indicating a slow polarization under an electric field, which was likely an indication that both dispersions followed the conduction model primarily rather than the polarization model. Similar conduction model behavior was reported for Fe_3_O_4_ in microporous covalent triazine-based polymer composites,^37^ while the polarization model was reported for the plain microporous covalent triazine-based polymer.^10^ Compared with the ER response of the microporous covalent triazine-based polymeric particles reported in the literature, our PTPA-COOH/SO system showed a much higher electric field response and better mechanical properties. Dong et al. found a yield stress of 156 Pa for 5% volume fraction microporous covalent triazine-based polymeric particles at 2 kV/mm electric field strength.^10^ Unfortunately, this comparison was not very useful, as the authors only worked at a single concentration, and only its rheological behavior was investigated up to an electric field strength of 2 kV/mm. There, unfortunately, was no discussion by the authors on why the effect of different concentrations or higher *E* was not studied.

**Figure 6:**
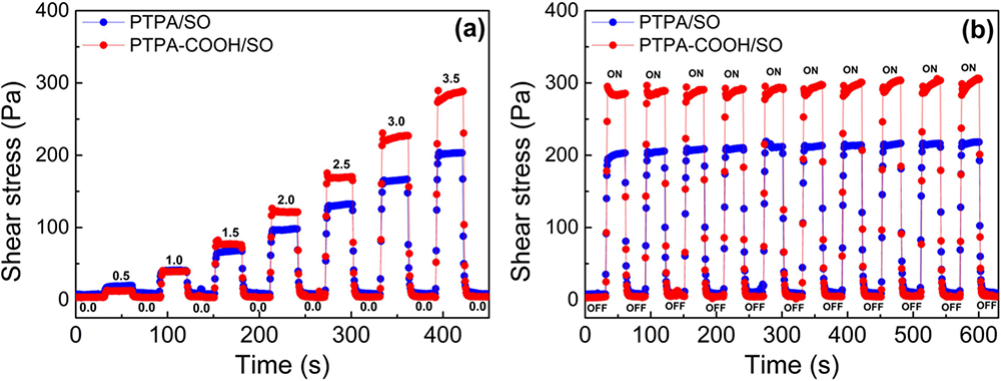
Change in shear stress of the PTPA/SO and PTPA-COOH/SO dispersions as a function of time in the on/off electric field mode alternately in (a) increasing and (b) constant (3.5 kV/mm) electric field strengths (γ̇ = 1 s^–1^, 10 wt %).

Reversible and fast electric field responses from a liquid-like to a solid-like state are other important characteristics of an excellent ER fluid for practical applications (in addition to possessing a strong ER performance). The responsiveness, reversibility, and long-term stability of the ER performance of the dispersions were tested by measuring the shear stress at a fixed shear rate of 1.0 s^–1^, while alternately turning the electric field on for 30 s and then off for 30 s in each switching cycle. The on–off electric field response curves of the CMP-based ER fluids are shown in Figure 6a,b. When the electric field was alternately turned on and off, the shear stress of the PTPA/SO and PTPA-COOH/SO dispersions increased instantaneously in the on-mode and dropped rapidly in the off-mode close to its initial value, indicating the excellent reversibility of the dispersions (Figure 6a). Consistent with the flow curves of the dispersions, the shear stresses of the dispersions increased with increasing *E*. When the dispersions were subjected to a constant square voltage pulse of 3.5 kV/mm at a fixed shear rate of 1 s^–1^ for 10 switching cycles, the dispersions maintained their durability, repeatability, and reversibility (Figure 6b). It should be noted that the shear stress hysteresis upon turning off the applied electric field was not significant, which indicated the reversible and rapid transition of the liquid-like to solid-like state upon application of *E* and desired well-destroyed field-induced structures after removal of *E*.^77^

Viscoelasticity is a property where materials show both viscous and elastic characteristics upon being subjected to deformation. Typically, ER fluids display primarily viscous behavior when an electric field is absent and primarily elastic behavior when the electric field is applied. The viscoelastic properties and phase transitions of ER fluids can be assessed through a dynamic oscillation test.^55^ To do so, initially, a stress or strain amplitude sweep test is conducted at a constant frequency value to identify the linear viscoelastic region (LVER), where stress and strain exhibit a proportional relationship.

**Figure 7:**
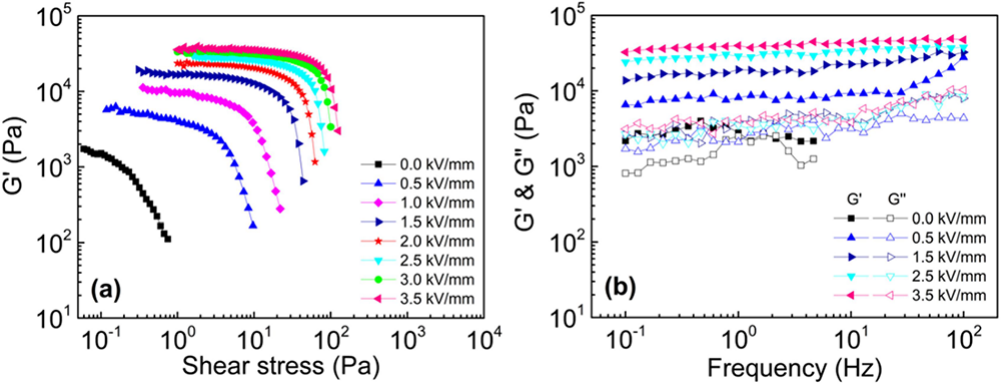
Change in (a) *G*′ with shear stress under various electric field strengths for PTPA-COOH/SO dispersion (*f* = 1 Hz) and (b) in *G*′ and *G*″ with increasing frequency in LVER for PTPA-COOH/SO dispersion (10 wt %).

The elastic (*G*′) and viscous (*G*″) moduli remain unaffected by the applied stress or strain, maintaining a plateau region in the low-stress or strain range. When the applied strain or stress is insufficient to cause structural breakdown of the field-induced structures, essential microstructural properties are evaluated.^78^ Beyond the LVER, nonlinearities emerge, complicating the directly related measurements of the microstructural properties. The limit of the LVER is called the critical stress (τ_c_). Figure 7a shows the variation in *G*′ with shear stress under various electric field strengths for the PTPA-COOH/SO dispersion. When stresses exceeding the τ_c_ were applied to the dispersion, the fibril-like structures broke down, resulting in a sharp decrease in the *G*′, and a nonrecoverable deformation was observed for all the *E* values. The τ_c_ of PTPA-COOH/SO is determined to be 0.07 Pa at *E* = 0 kV mm^–1^ and 12 Pa at *E* = 3.5 kV mm^–1^. Notably, the τ_c_ increased and the LVER broadened because of the increased quantity of electrostatic interactions among the dispersed particles induced by the electric field.

An angular frequency sweep test was conducted within the predefined LVER for the dispersions, analyzing its time-dependent behavior in the nondestructive deformation range. This test aimed to investigate the behavior and internal structure, the influence of colloidal forces, particle interactions, and the ER fluid’s long-term stability.^79^ In the electric field “off” state, *G*′ < *G*″ is typically observed at low frequencies, indicating a predominance of liquid-like behavior. Subsequently, the crossover point of *G*′ and *G*″ emerges, associated with the relaxation time. In the high-frequency region, *G*′ > *G*″ is displayed, indicating a predominance of solid-like behavior for viscoelastic liquids.

Moreover, within the LVER, *G*′ may exhibit minimal dependence on frequency, consistent with the behavior anticipated in a structured or solid-like material. The elasticity of the material becomes more fluid-like as the dependence of the elastic modulus on frequency increases. Figure 7b indicates the change in *G*′ and *G*″ with increasing frequency in the LVER. Under no electric field, the PTPA-COOH/SO dispersion with 10 wt % concentration exhibited almost no crossover frequency, and the storage modulus consistently surpassed the loss modulus (*G*′ > *G*″) across the entire frequency range studied, indicative of gel-like behavior. This behavior could be ascribed to the increased attractive physical particle–particle interactions with the aid of higher particle concentrations and the formation of secondary bridging within the dispersed particles. Upon application of the electric field, *G*′ significantly exceeded *G*″, and *G*′ and *G*″ remained parallel across the entire frequency range, indicating a stable gel-like structure with solid-like behavior. As *E* increased, the modulus values also increased, indicating enhanced structural strength. The application of *E* = 3.5 kV mm^–1^ resulted in a 14-fold increase in the elastic modulus compared to the absence of the *E*. Additionally, the constant and dominant elastic modulus indicated the potential use of these dispersions in vibration-damping systems, which depend on storing the applied force and turning it into heat during operations. The formation of fibril-like structures under an electric field enhances the presence of viscous friction forces, enabling the storage of applied energy until eventual dissipation.

## 4 Conclusions

To explore the potential of three-dimensional analogues of the well-known conducting polymer PAni for application as active smart materials in ER fluids, PTPA and the extended carboxylic acid functionalized version, PTPA-COOH, were synthesized by Buchwald–Hartwig cross-coupling. The ER responses of up to 10 wt % dispersions of these materials in SO were successfully explored, which not only enabled comparison of these two materials with other published systems but also highlighted the promise of our approach: the combination of hierarchical porosity and the presence of highly polarizable −COOH groups in the CMP structure significantly enhanced the ER performance of PTPA-COOH compared with the unfunctionalized version and other PAni-related systems. Notably, the more polar surface and improved hierarchical porous structure played a key role in enhancing dispersion stability, rapid achievable polarization, and repeatable electric field response of PTPA-COOH. This research not only broadens the application of CMPs beyond traditional gas capture and energy storage but also provides insight and understanding of the key roles that surface polar groups and porosity play in the design and synthesis of ER-active materials. Further approaches for enhanced electroresponsive fluid applications are therefore envisaged in the near future.

## Supporting Information Available

Mechanism of the Buchwald–Hartwig amination (Scheme S1), contact angle measurements for PTPA (a) and PTPA-COOH (b) at 0 and 5 min of dropping water (Figure S1), the optimal parameters in the CCJ model equation obtained from the flow curves of PTPA/SO and PTPA-COOH/SO dispersions (10 wt %) at various electric field strengths (Table S1), and shear rate vs shear stress curves recorded in CSS mode to determine the static yield stress of PTPA/SO (Figure S2) and PTPA-COOH/SO (Figure S3) dispersions with various concentrations under various electric field strengths (PDF)
