# Supplementary material for: Electrorheological Fluids Based on Porous Carboxyl-Functionalized Polytriphenylamines
Source: ACS Appl Polym Mater. 2025 Jan 25;7(3):1205–16. doi: 10.1021/acsapm.4c02469 (PMC11833765; doi:10.1021/acsapm.4c02469)
Supplement: Supplementary file 1 — ap4c02469_si_001.pdf [file ap4c02469_si_001.pdf]

Supporting Information

Electrorheological Fluids Based on Porous Carboxyl-Functionalized Polytriphenylamines

Ozlem Ero1,[1‡]* Ulzhalgas Karatayeva*[2‡] *and Charl F. J. Faul*[2]*

[1]Chemistry Department, Science Faculty, Gazi University, 06560 Ankara, Turkey

[2]School of Chemistry, University of Bristol, Bristol BS8 1TS, U.K.

‡These authors contributed equally to this work.

*Corresponding authors

*E-mail: oerol@gazi.edu.tr; charl.faul@bristol.ac.uk

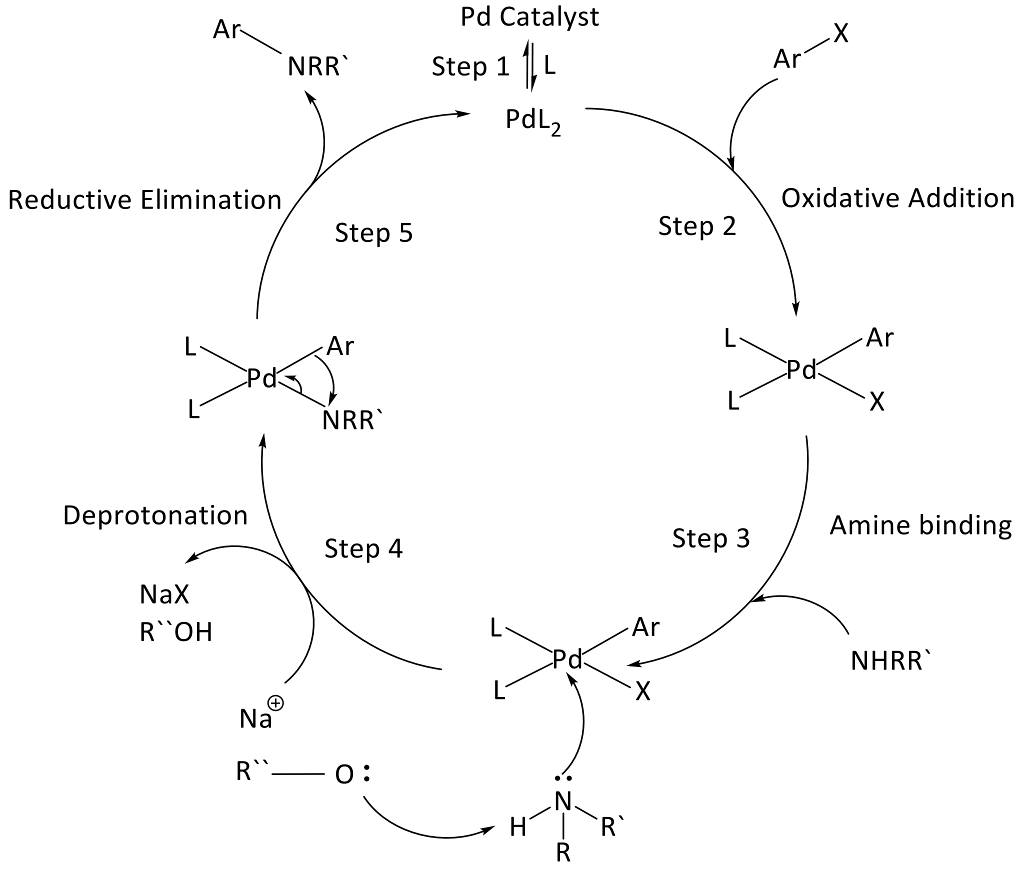

Scheme S1. Mechanism of the Buchwald-Hartwig amination.

Scheme S1 illustrates the reaction mechanism of Buchwald-Hartwig amination, which proceeds as follows. Initially, the catalytic cycle commences with the formation of a complex between the Pd catalyst and ligand (L) (Step 1). Subsequently, the aryl halide (Ar-X) undergoes oxidative addition to the Pd-ligand complex (PdL_2) (Step 2), resulting in an elevation of the oxidation state of Pd (from Pd(0) to Pd(II)). Next, the amine (NHRR`) binds to the complex (Step 3), succeeded by the addition of a base (NaOR``) to eliminate the halide through deprotonation, yielding an amide (Step 4). Finally, reductive elimination occurs (Step 5), facilitating the formation of new strong covalent bonds to produce the amination product (Ar-NRR`), concurrently reducing the oxidation state of Pd and regenerating the catalyst for subsequent cycles.[1]

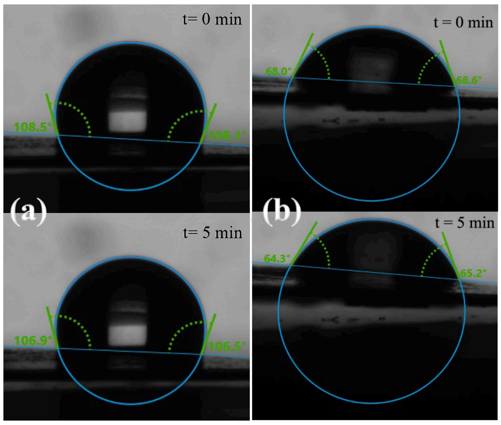

Figure S1. Contact angle measurements for PTPA (a) and PTPA-COOH (b) at 0 min and 5 min of dropping water.

Table S1. The optimal parameters in the CCJ model equation obtained from the flow curves of PTPA/SO and PTPA-COOH/SO dispersions (10 wt%) at various electric field strengths.

**Sample**	**Parameter**	**Electric field strength (kV/mm)**						
		**0.5**	**1.0**	**1.5**	**2.0**	**2.5**	**3.0**	**3.5**
**PTPA/SO**	τ_{dy}	14.5	37.0	65.0	98.0	129.0	163.0	200.0
	t_1	0.05	0.03	0.022	0.01	0.015	0.015	0.01
	t_2	0.0008	0.0007	0.0007	0.0007	0.0009	0.0009	0.00095
	α	1.6	2.2	2.2	2.2	2.2	2.2	2.5
	β	0.2	0.18	0.18	0.18	0.25	0.25	0.25
	η_∞	1.4	1.4	1.4	1.4	1.45	1.5	1.5
**PTPA-COOH/SO**	τ_{dy}	17	60	108	165	226	293	350
	t_1	0.5	0.28	0.1	0.05	0.005	0.003	0.0007
	t_2	0.06	0.05	0.028	0.035	0.5	0.2	0.25
	α	0.70	0.56	0.45	0.45	0.25	0.22	0.24
	β	0.20	0.25	0.23	0.23	0.3	0.36	0.36
	η_∞	1.2	1.2	1.2	1.2	1.4	1.4	1.45

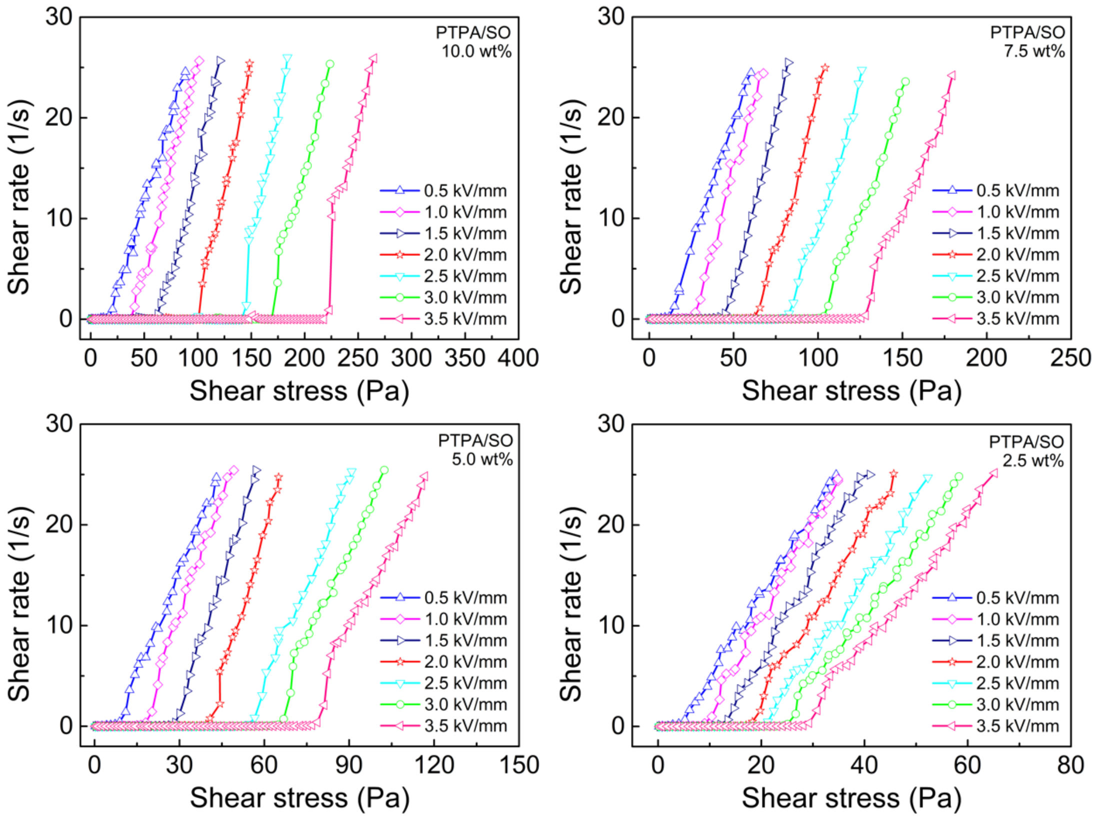

Figure S2. Shear rate vs shear stress curves recorded in CSS mode to determine the static yield stress of PTPA/SO dispersions with various concentrations under various electric field strengths.

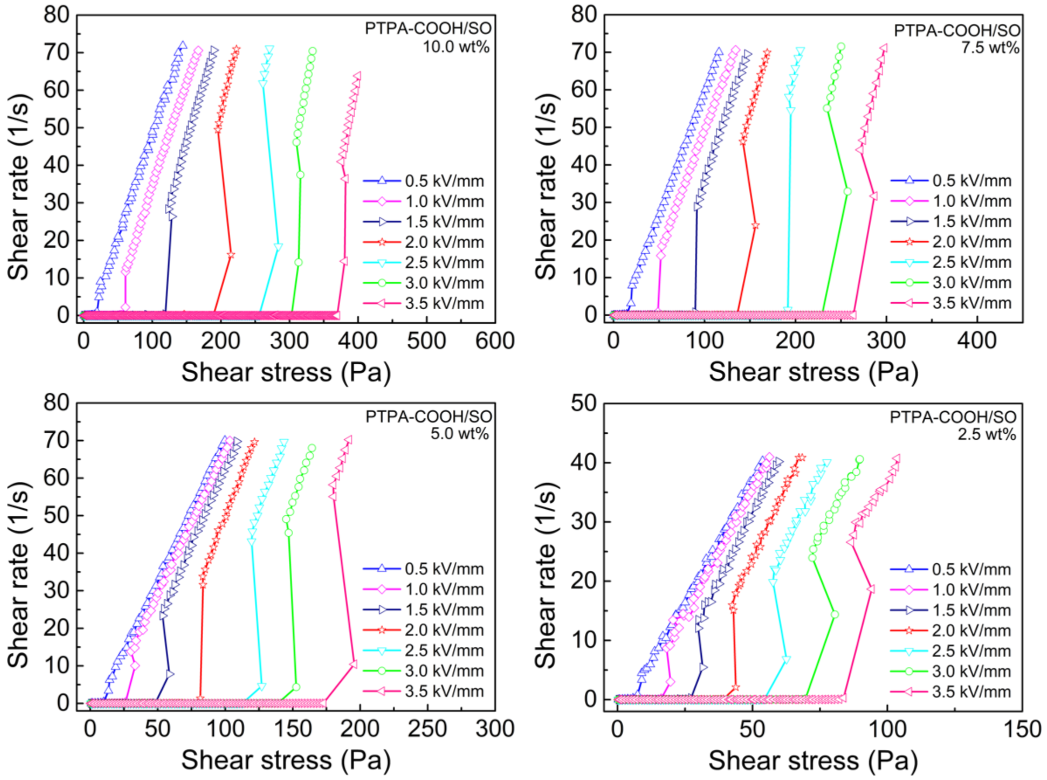

Figure S3. Shear rate vs shear stress curves recorded in CSS mode to determine the static yield stress of PTPA-COOH/SO dispersions with various concentrations under various electric field strengths.

REFERENCES

(1) Wambua V.; Hirschi J. S.; Vetticatt M. J. Rapid Evaluation of the Mechanism of Buchwald-Hartwig Amination and Aldol Reactions Using Intramolecular ^{13}C Kinetic Isotope Effects. *ACS Catalysis*, **2021**, *11*, 60–67. DOI: 10.1021/acscatal.0c04752.
